# Efficacy, safety and tolerability of very low-calorie ketogenic diet in obese women with fibromyalgia: a pilot interventional study

**DOI:** 10.3389/fnut.2023.1219321

**Published:** 2023-07-12

**Authors:** Jacopo Ciaffi, Lucia Lisi, Anna Mari, Luana Mancarella, Veronica Brusi, Federica Pignatti, Susanna Ricci, Giorgia Vitali, Nicola Stefanelli, Elisa Assirelli, Simona Neri, Susanna Naldi, Cesare Faldini, Francesco Ursini

**Affiliations:** ^1^Medicine & Rheumatology Unit, IRCCS Istituto Ortopedico Rizzoli, Bologna, Italy; ^2^Dietetic Service, IRCCS Istituto Ortopedico Rizzoli, Bologna, Italy; ^3^1st Orthopaedic and Traumatology Department, IRCCS Istituto Ortopedico Rizzoli, Bologna, Italy; ^4^Department of Biomedical and Neuromotor Sciences (DIBINEM), University of Bologna, Bologna, Italy

**Keywords:** fibromyalgia, ketone bodies, pain, obesity, ketogenic diet

## Abstract

**Introduction:**

Obesity can worsen fibromyalgia (FM) and very low-calorie ketogenic diet (VLCKD) is a potential therapeutic option for diseases that share clinical and pathophysiological features with FM. In this pilot interventional study, we investigated the effects of VLCKD in obese women with FM.

**Methods:**

Female patients with FM and a body mass index (BMI) ≥ 30  kg/m^2^ were eligible for VLCKD. The ketogenic phase (T0 to T8) was followed by progressive reintroduction of carbohydrates (T8 to T20). Changes in BMI, Fibromyalgia Impact Questionnaire (FIQ), Hospital Anxiety and Depression Scale (HADS), EuroQol 5D (EQ-5D) and 36-item Short Form Health Survey (SF-36) were evaluated. A change of 14% in FIQ was considered clinically relevant. The longitudinal association between BMI and patient-reported outcomes (PROs) was assessed using generalized estimating equations.

**Results:**

Twenty women were enrolled. Two discontinued the intervention. The mean age of the 18 patients who reached T20 was 51.3  years and mean BMI was 37.2  kg/m^2^. All patients lost weight during the first period of VLCKD and this achievement was maintained at T20. Mean BMI decreased from 37.2  kg/m^2^ at T0 to 34.8  kg/m^2^ at T4, 33.5  kg/m^2^ at T8 and 32.1  kg/m^2^ at T20 (*p* < 0.001). A significant reduction of mean FIQ from 61.7 at T0 to 37.0 at T4 and to 38.7 at T8 (*p* < 0.001) was observed and it was maintained at T20 with a mean FIQ of 39.1 (*p* = 0.002). Similar results were obtained for HADS, EQ-5D and SF-36. Analysing each participant, the reduction of FIQ was clinically meaningful in 16 patients (89%) at T4, in 13 (72%) at T8 and in 14 (78%) at T20. No significant association was observed between change in BMI and improvement of the PROs over time. Adverse effects were mild and transient. No major safety concerns emerged.

**Conclusion:**

These are the first data on the efficacy of VLCKD in FM. All patients achieved improvement in different domains of the disease, which was maintained also after carbohydrate reintroduction. Our results suggest that ketosis might exert beneficial effects in FM beyond the rapid weight loss.

**Clinical trial registration:**

This trial is registered on ClinicalTrials.gov, number NCT05848544.

## 1. Introduction

Fibromyalgia (FM) is a chronic disorder characterized by widespread pain, sleep problems, fatigue and cognitive impairment (1). Although its pathogenesis remains incompletely understood, central, peripheral and cognitive emotional sensitization mechanisms can be involved in the nociplastic process (2–4). In addition, widespread pain is associated with body fat mass (5) and longitudinal data suggest that being obese or overweight constitutes a significant risk factor for developing FM over time (6, 7). Robust evidence describes the association between obesity and FM, with a recent systematic review reporting a prevalence of obesity close to 36% in FM patients (8), but the role of weight loss in relieving FM symptoms has not been fully elucidated. The available literature provides preliminary support that weight reduction through hypocaloric diet or bariatric surgery can improve the severity of symptoms and quality of life in patients with FM (9–15). In the last few years, a novel nutritional approach – namely Very Low Calorie Ketogenic Diet (VLCKD) – has been proposed as a potential therapeutic option in different diseases that share common clinical and pathophysiological features with FM (16). Originally conceived to treat epilepsy before the introduction of antiepileptic drugs, the concept of inducing the production of ketone bodies through a low-carbohydrate diet with the purpose of improving patients’ symptoms has been applied to neurodegenerative diseases, musculoskeletal disorders and oncological conditions (17–20). VLCKD proved to be effective in achieving rapid weight loss in obese subjects who failed other dietetic interventions and it was included as a therapeutic option in the guidelines for the management of obesity (21, 22). Furthermore, accumulating evidence suggests positive effects on mood, cognitive functions, nociception and sleep quality (23–26), but no data are available about the effects of VLCKD in patients with FM. Therefore, we conducted a pilot interventional study to determine whether VLCKD could be safe, well-tolerated and effective in improving the disease burden in obese women with FM.

## 2. Materials and methods

### 2.1. Study design and participants

The trial was conducted at IRCCS Istituto Ortopedico Rizzoli, Bologna, Italy from January 2022 to March 2023. Adult patients with FM presenting to the Rheumatology outpatient clinic were considered for inclusion. Given the exploratory nature of the study, the strong female preponderance of the disease in Italy and the different characteristics of pain and response to therapy between men and women (27, 28), we predefined to enrol an arbitrary number of 20 patients, all females. Patients were eligible to VLCKD according to the Italian Standards for Treatment of Obesity, released by the Italian Society for the Study of Obesity and the Italian Association of Dietetics and Clinical Nutrition (2016–2017) and the recommendations of the Italian Society of Endocrinology for VLCKD (22). Women between age 18 and 65 years with a body mass index (BMI) ≥ 30  kg/m^2^ and a diagnosis of FM fulfilling the 2010/2011 American College of Rheumatology (ACR) criteria (29) were considered for inclusion if they had a history of failure to lose weight with standard hypocaloric diets. The presence of at least one of the following conditions of cardiometabolic risk was required: BMI ≥ 35 kg/m^2^; past diagnosis of type 2 diabetes without β-cell failure; hypertriglyceridemia (fasting triglycerides ≥150 mg/dL); hypercholesterolemia (total cholesterol >200 mg/dL) or taking lipid-lowering medications; past diagnosis of arterial hypertension or taking blood pressure-lowering medications; past diagnosis of non-alcoholic fatty liver disease; past diagnosis of heart failure New York Heart Association (NYHA) class I–II; past history of myocardial infarction (> 12 months) or stroke/minor stroke (>12 months); past diagnosis of carotid atherosclerosis; past diagnosis of polycystic ovary syndrome; past diagnosis of neurodegenerative disorders.

Exclusion criteria were: pregnancy or breastfeeding; past diagnosis of type 1 diabetes, latent autoimmune diabetes in adults, β-cell failure in type 2 diabetes or use of sodium/glucose cotransporter 2 (SGLT2) inhibitors; past diagnosis of kidney failure and moderate-to-severe chronic kidney disease; liver failure; hearth failure NYHA class III-IV; respiratory failure; past diagnosis of unstable angina; recent stroke or myocardial infarction (<12 months); cardiac arrhythmias; past diagnosis of eating disorders and other severe mental illnesses; alcohol and substance abuse; active/severe infections; past diagnosis of rare disorders; allergy to the ingredients of the protein-preparations; past or current history of gallstones.

### 2.2. Dietary intervention

**Figure 1:**
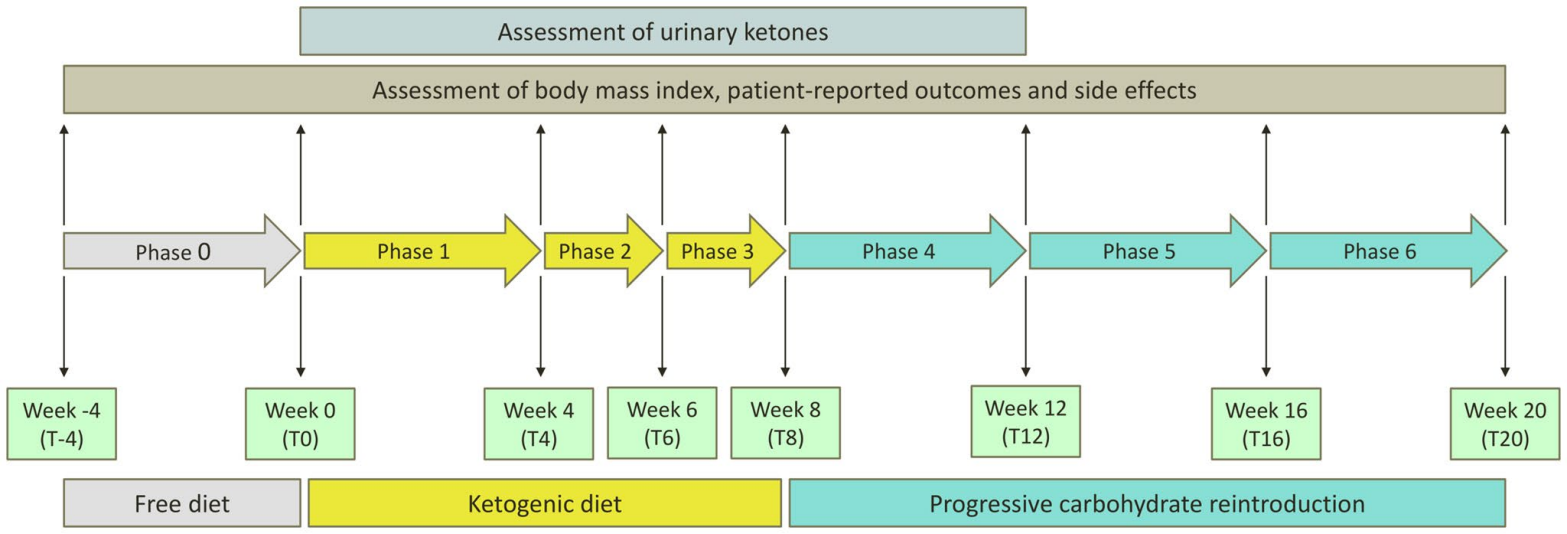
Flowchart of the study protocol. A 4 weeks run-in period of free diet preceded the weight-loss program (Phase 0 – T-4 to T0). The ketogenic period was divided into three phases. During the first 4 weeks (Phase 1 – T0 to T4), patients were allowed to eat 4 to 6 protein preparations every day with an average daily caloric intake of 801 kcal. The next 4 weeks were divided into two 2 weeks periods (Phase 2 – T4 to T6 and Phase 3 – T6 to T8), in which one (Phase 2) or two (Phase 3) of the meal preparations were replaced by natural proteins, for an average daily caloric intake of 843 kcal. Carbohydrates were then progressively reintroduced, starting from foods with low glycemic index during the next 4 weeks (Phase 4 – T8 to T12) and then continuing with moderate (Phase 5 – T12 to T16) and high (Phase 6 – T16 to T20) glycemic index products. The average daily caloric intake gradually increased from 1,138 kcal in the fourth phase to 1,186 kcal in the fifth phase and 1,490 kcal in the sixth phase. The presence of ketosis was assessed at weekly intervals from T0 to T8 and then at T12 using urine strips.

**Figure 2:**
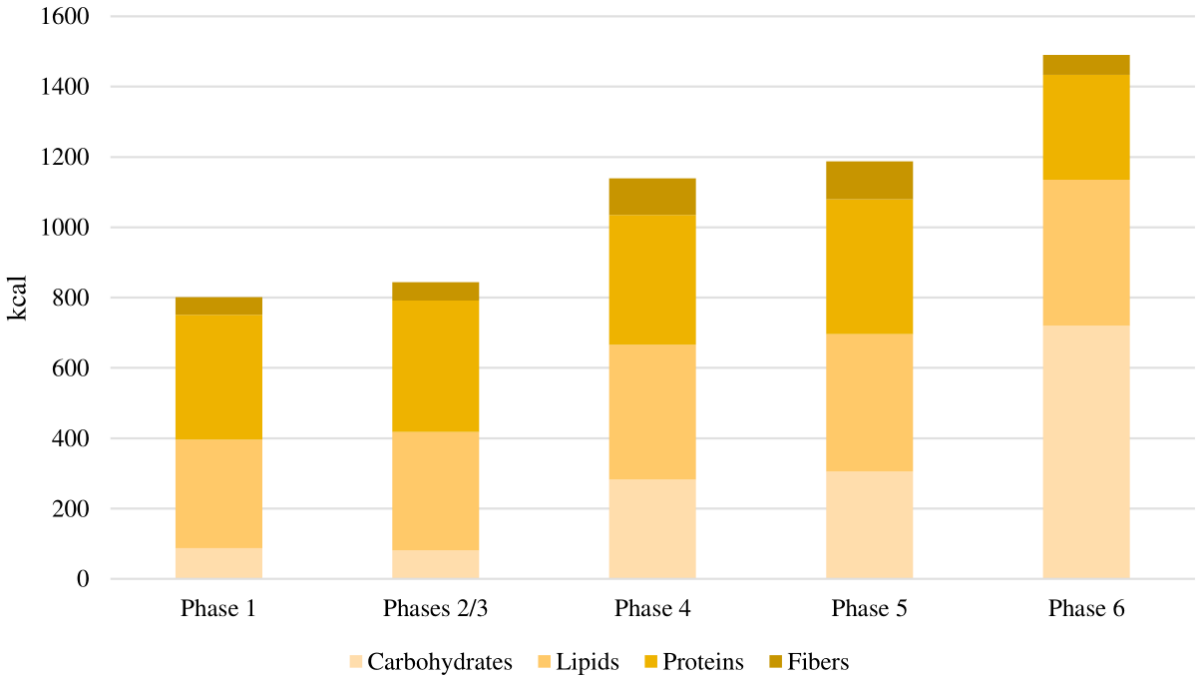
Average daily caloric intake for each phase of the study. In the first phase, the average daily caloric intake was 801 kcal, with a macronutrient ratio of 11% carbohydrates, 39% fat, 44% proteins and 6% fibers. In the second and third phases, the average daily caloric intake was 843 kcal, with a macronutrient ratio of 10% carbohydrates, 40% fat, 44% proteins and 6% fibers. In the fourth phase, the average daily caloric intake was 1,138 kcal, with a macronutrient ratio of 25% carbohydrates, 34% fat, 32% proteins and 9% fibers. In the fifth phase, the average daily caloric intake was 1,186 kcal, with a macronutrient ratio of 26% carbohydrates, 33% fat, 32% proteins and 9% fibers. In the sixth phase, the average daily caloric intake was 1,490 kcal, with a macronutrient ratio of 48% carbohydrates, 28% fat, 20% proteins and 4% fibers.

The flowchart of the study protocol is shown in Figure 1. During the screening visit, eligible patients attended a consultation with a rheumatologist and two dietitians. They were informed about the principles and the practical aspects of the VLCKD and they were shown how to use a food diary and a urine ketone testing strip. Demographic data and medical history were collected. Before the weight-loss program, enrolled patients underwent a 4 weeks run-in period. During this free-diet phase (Phase 0 – T-4 to T0), patients were invited to eat normal meals and to complete a daily food record for 2 consecutive weeks. Personalized diet plans were then developed for each participant based on their food preferences using a combination of commercially available ketogenic preparations and handmade meals. The commercial preparations were provided by an authorized supplier on the basis of individual choices and delivered to patients by a dietician investigator at the beginning of the ketogenic period. Additionally, the free-diet phase provided a period to be used for the self-controlled design of the study. The ketogenic period was divided into three phases. During the first 4 weeks (Phase 1 – T0 to T4), patients were allowed to eat 4 to 6 protein preparations every day with different recipe options and low-carbohydrate vegetables. In this phase, each portion of meal preparation contained 25 to 50 grams of dry product providing 90 to 205 kcal with a protein content ranging from 30 to 72%. It was also recommended to drink not less than 2 litres of water or clear liquids (tea, coffee, unsweetened carbonated drinks) per day. The average daily caloric intake for each phase of the study is shown in Figure 2. In the first phase, it was 801 kcal, with a macronutrient ratio of 11% carbohydrates, 39% fat (4% saturated), 44% proteins and 6% fibers. The next 4 weeks were divided into two 2 weeks periods (Phase 2 – T4 to T6 and Phase 3 – T6 to T8). The state of ketosis was still maintained, but one (Phase 2) or two (Phase 3) of the provided meal preparations were replaced by natural proteins (meat, fish, eggs or legumes). In the second and third phases, the average daily caloric intake was 843 kcal, with a macronutrient ratio of 10% carbohydrates, 40% fat (5% saturated), 44% proteins and 6% fibers.

Following the ketogenic period, carbohydrates were progressively reintroduced, starting from foods with low glycemic index during the next 4 weeks (Phase 4 – T8 to T12) and then continuing with moderate (Phase 5 – T12 to T16) and high (Phase 6 – T16 to T20) glycemic index products, allowing for a gradual nutritional education and strengthening the long-term weight-loss achievements. In the fourth phase, the average daily caloric intake was 1,138 kcal, with a macronutrient ratio of 25% carbohydrates, 34% fat (4% saturated), 32% proteins and 9% fibers. In the fifth phase, the average daily caloric intake was 1,186 kcal, with a macronutrient ratio of 26% carbohydrates, 33% fat (4% saturated), 32% proteins and 9% fibers. In the sixth phase, the average daily caloric intake was 1,490 kcal, with a macronutrient ratio of 48% carbohydrates, 28% fat (5% saturated), 20% proteins and 4% fibers.

No changes in the dosages of antidepressants, anticonvulsants, hypnotics, muscle relaxants, or antipsychotics were allowed during the study period. On demand analgesic drugs could be continued. Additionally, patients were advised to avoid making major changes to their usual activities regarding fitness, psychotherapy, mindfulness, acupuncture, or any other non-pharmacological treatment.

### 2.3. Adherence

Patients were asked to monitor urinary ketosis using the urine strips provided at the time of enrolment (Multistix 10SG, Siemens Healthcare Diagnostics, Inc., Tarrytown NY). The presence of ketosis was assessed at weekly intervals from T0 to T8 and then at T12. Patients were instructed about the correct usage of the strips and to send a picture of the results to a dedicated email address for evaluation by one of the investigators. During the study period, patients were not allowed to use medications potentially responsible for unreliable urinary ketone results such as valproic acid, captopril, levodopa, ascorbic acid or nitrates (30–32).

### 2.4. Safety

Safety was monitored through regular blood and urine tests taken before the beginning of VLCKD, every 4 weeks during the ketogenic phase and then at study completion. Each patient was instructed to maintain a daily log of meals and of dietary intolerance symptoms. Any presumed adverse event could be communicated using the phone number provided to all patients and their families. Diet tolerance and adverse events were further evaluated during the study visits. Adverse events were systematically monitored. In case of adverse events or intolerance, the possibility to withdraw from the study was discussed between the patient and the investigators. In women of childbearing age, rapid pregnancy test was obtained before starting VLCKD. Patients were instructed to immediately communicate to the study staff the event of an unexpected pregnancy during the ketogenic phase and to stop the diet.

### 2.5. Study outcomes

Assessment of disease activity was performed using validated self-administered tools (patient-reported outcomes, PROs) as suggested by the international Outcome Measures in Rheumatology Clinical Trials (OMERACT) working group proposal for Fibromyalgia Responder Index and Disease Activity Score (33), including Fibromyalgia Impact Questionnaire (FIQ) (34), Hospital Anxiety and Depression Scale (HADS) (35), EuroQoL 5 Dimensions 3 Levels (EQ-5D) (36) and 36-item Short Form Health Survey (SF-36) (37). PROs and body weight measurements were obtained at T-4, T0, T4, T8, T12, T16 and T20.

#### 2.5.1. Fibromyalgia impact questionnaire

The FIQ is among the most widely used tools in research and clinical practice to evaluate the impact of FM on patients’ health status (34, 38). The questionnaire is based on recall in the past week and it is composed of 10 items assessing physical function, workplace absenteeism, occupational impairment, pain, fatigue, morning tiredness, stiffness, anxiety and depression. The overall score ranges from 0 to 100, with higher values indicating a greater impact of the disease. Previous literature suggests that a change of 14% in the total score can be considered a minimum clinically important difference (MCID) (39). The English version of the FIQ is shown in Supplementary File 1 (38).

#### 2.5.2. Hospital anxiety and depression scale

The Hospital Anxiety and Depression Scale (HADS) was originally developed to measure anxiety and depression in a general population of medical patients (35). The questionnaire comprises 14 items scored from 0 to 3 with 7 questions for anxiety and 7 for depression, which are summarised into two independent scores, HADS-A and HADS-D. For each domain, the total score ranges from 0 to 21. A cut-off of >10 has been suggested to distinguish between cases and non-cases in non-clinical populations (40). The English version of the HADS is shown in Supplementary File 2 (41).

#### 2.5.3. EuroQoL-5 dimensions

The EQ-5D can be used as a generic preference-based questionnaire to measure health status (36). The questionnaire consists of two distinct parts. The first part is a descriptive system comprising 5 domains: mobility, self-care, usual activities, pain/discomfort, and anxiety/depression. For each dimension, patients can choose between three severity levels: no problems, some problems and extreme problems. Responses can be converted into a single sum utility score using preference-based nation-specific weights (42). The Italian tariff was applied in this study and the results vary from −0.39 to 1 (43). Negative scores indicate a patient’s perception of health status worse than death, while a score of 1 indicates perfect health. The second part of the questionnaire consists of a thermometer-like visual analogue scale (VAS) through which patients are asked to rate their health of the day from 0 (worst imaginable health) to 100 (best imaginable health). The English version of the EQ-5D is shown in Supplementary File 3 (44).

#### 2.5.4. 36-item short form health survey

The 36-item Short Form Health Survey (SF-36) is a generic multidimensional index used to evaluate self-perception of quality of life and health status through physical and mental functioning (37). The instrument assesses eight areas: physical function, physical role, bodily pain, general health, vitality, social function, emotional role and mental health. A score of 0 represents poor health status, while 100 represents good health status. The Italian version of the questionnaire was used in this study (45). The results were summarised into two global scores, namely the physical component score (PCS) and the mental component score (MCS). The English version of the SF-36 is shown in Supplementary File 4 (46).

### 2.6. Statistical analysis

Data are expressed as mean ± standard deviation or median (25th – 75th quartile) or number (percentage), as appropriate. Paired samples Student’s *t*-test was used to compare differences in BMI, FIQ, HADS, EQ-5D and SF-36 across the study time points. Generalized estimating equations (GEEs) were used to analyse the association over time between BMI and the PROs included in the study. Independent longitudinal models with linear response and autoregressive correlation structure were built. All models were adjusted for age and disease duration, with either FIQ, HADS-A, HADS-D, EQ-5D utility score or VAS, SF-36 MCS or SF-36 PCS as dependent variables. Data were analyzed through a per-protocol approach from patients who achieved ketosis during the first 8 weeks of the dietary intervention and completed both the ketogenic and carbohydrate reintroduction phases of the study. A value of *p* <0.05 was considered statistically significant. All analyses were performed using the Statistical Package for Social Sciences (SPSS) software version 28.0 (IBM).

### 2.7. Ethical considerations

The research was conducted in compliance with the Declaration of Helsinki and its latest amendments (47). The study protocol was approved by the local Ethics Committee (Comitato Etico Area Vasta Emilia Centrale, Bologna, Italy – approval number: 0017502/2021) and written informed consent was obtained from all participants. This trial is registered on ClinicalTrials.gov, number NCT05848544.

## 3. Results

### 3.1. Characteristics of patients

**Figure 3:**
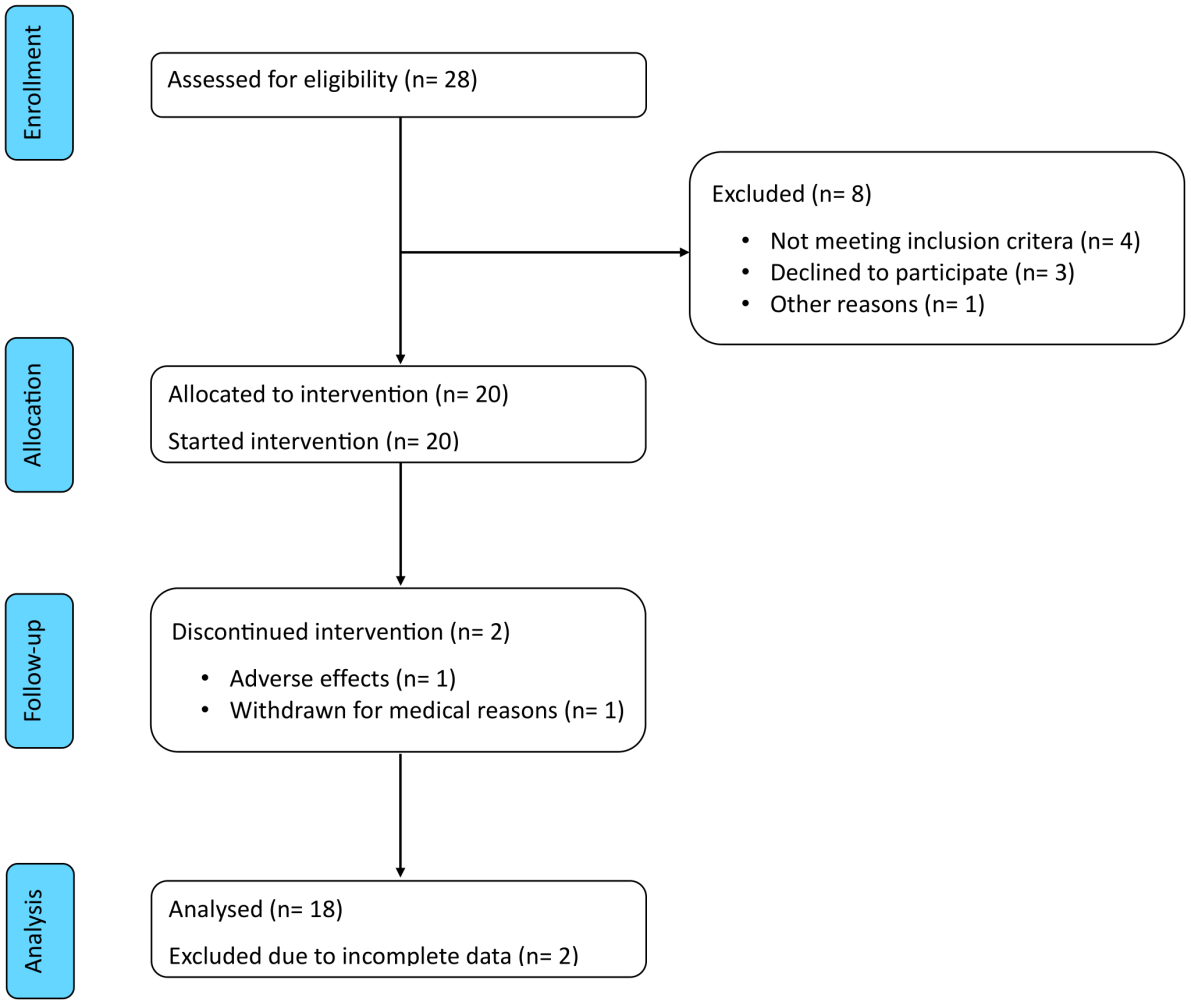
CONSORT flow diagram. Of the 28 patients assessed for eligibility, 8 were excluded and 20 were enrolled in the study. Of these, 2 discontinued the intervention and 18 completed the study period and were included in the analysis.

Eligibility was assessed in 28 patients to include the predefined number of 20 participants. Of these, 18 (90%) completed both the 8 weeks of ketogenic diet and the subsequent 12 weeks of carbohydrate reintroduction. The Consolidated Standards of Reporting Trials (CONSORT) flow diagram is shown in Figure 3. The characteristics of these patients are summarised in Table 1. Mean age was 51.3 ± 9.5 years and median disease duration was 3.6 (IQR 1.6–5.3) years. Mean BMI was 37.2 ± 9.5 kg/m^2^ and 13 patients (72%) had a BMI >35 kg/m^2^. Among the comorbidities, hyperlipidaemia (*n* = 13, 72%) and hypertension (*n* = 11, 61%) were the most frequently represented, followed by non-alcoholic fatty liver disease (*n* = 8, 44%) and polycystic ovary syndrome (*n* = 2, 11%), while no patient was diabetic or had a history of atherothrombotic events or congestive heart failure.

**Table 1:** Baseline characteristics of patients who completed the study.

Characteristics	Patients (*n* = 18)
Age, years	51.3 ± 9.5
Disease duration, years	3.6 (1.6–5.3)
BMI, Kg/m^2^	37.2 ± 3.8
BMI > 35	13 (72)
Comorbidities	
Hypertension	11 (61)
Hyperlipidemia	13 (72)
Non-alcoholic fatty liver disease	8 (44)
Polycystic ovary syndrome	2 (11)
Type 2 diabetes	0
Previous atherothrombotic events	0
Congestive heart failure	0
Neurodegenerative disorders	0
Medications	
Non-steroidal anti-inflammatory drugs	8 (44)
Paracetamol	7 (39)
Antidepressant	5 (28)
Gabapentinoids	3 (17)
Benzodiazepines	3 (17)
Muscle relaxants	1 (6)
Patient-reported outcomes	
FIQ	61.7 ± 22.2
HADS-A	11.1 ± 4.0
HADS-D	9.6 ± 3.7
EQ-5D utility score	0.6 ± 0.2
EQ-5D VAS	42.0 ± 23.7
SF-36 MCS	34.3 ± 16.7
SF-36 PCS	30.3 ± 14.8

BMI, body mass index; EQ-5D, EuroQoL 5 dimensions; FIQ, fibromyalgia impact questionnaire; HADS-A, hospital anxiety and depression scale-anxiety; HADS-D, hospital anxiety and depression scale-depression; MCS, mental component score; PCS, physical component score; SF-36, 36-item short form health survey; VAS, visual analogue scale.

At the baseline visit, 8 patients (44%) reported to use non-steroidal anti-inflammatory drugs (NSAIDs), while 7 (39%) managed their pain with paracetamol and 2 (11%) with opioids. Five patients (28%) were treated with antidepressants, 3 (17%) with gabapentinoids, 3 (17%) with benzodiazepines and 1 (6%) with muscle relaxants.

### 3.2. Adherence to ketogenic diet

Urine ketone assessments demonstrated that each patient achieved ketosis although the degree and consistency of ketosis were variable. As shown in Table 2, urine ketones were not found in any participant before the beginning of the ketogenic phase, whereas between weeks 1 and 8, ketones were detected in 2 or more samples in all patients. In particular, urinary ketones were found 2 times in 1 patient (6%), 5 times in 3 patients (17%), 6 times in 2 patients (11%) and 7 times in 3 patients (17%). In half of the cases, ketones were found in every determination. Furthermore, 5 participants also had urinary ketones at T12, the end of the low-glycemic index carbohydrates reintroduction phase.

**Table 2:** Assessment of urinary ketones.

Patient	Timepoint of the study									
	T0	T1	T2	T3	T4	T5	T6	T7	T8	T12
FM1			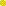			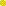	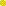	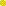	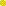	
FM2		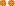	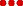	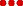	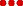	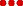	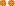	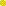		
FM3			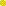	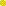						
FM4		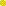	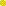	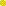	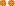	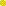	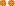	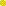	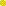	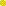
FM5			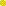	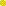	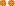	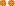	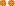	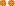	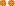	
FM6		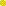	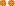	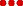	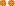	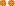	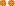	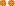	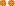	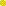
FM9		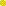	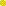	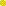	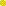	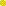	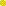	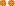	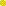	
FM10			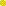	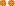	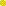	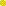	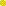	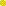		
FM11		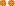	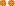	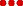	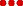	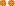	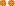	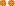	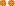	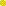
FM12		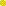	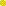	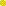	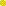	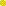	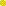	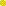	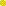	
FM13		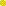	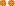	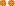	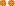	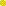	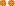	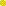	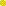	
FM14			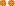	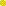	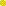	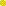	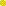	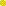	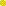	
FM15		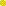	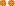	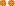	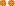	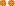	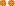	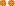	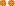	
FM16		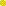	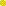	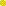	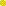	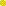	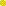	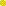	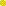	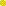
FM17		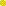	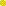	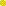	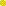	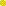	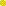	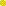		
FM18		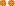	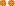	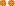	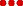	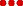	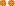	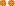	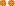	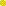
FM19					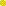	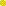	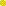	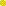	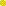	
FM20				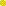	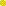	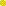	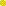	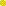		

Amount of ketones in the urine: absent, 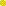 small, 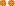 moderate, 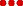 large.

### 3.3. Safety

VLCKD was well tolerated. There was one dropout after the second week of the ketogenic phase due to worsening of pre-existing headache and refusal from the participant to further alter her diet. The other patient who did not complete the study period was withdrawn from the trial before the end of the ketogenic phase when an abdominal ultrasound, requested for persistent abdominal discomfort, revealed liver lesions. The patient was referred to the oncology department and additional workup led to the diagnosis of metastatic breast cancer.

The participants who completed the 20-weeks intervention reported only mild and transient adverse events, which were noted among 12 of 18 patients (67%). The commonest adverse event was constipation (*n* = 11, 61%), followed by fatigue (*n* = 6, 33%), while 4 patients (22%) complained about headache and 3 (17%) about bloating. Diarrhoea and abdominal discomfort were each described by one patient (6%). No safety concerns emerged from laboratory tests during the ketogenic phase or at the end of the intervention.

### 3.4. Efficacy

#### 3.4.1. Change in BMI

**Figure 4:**
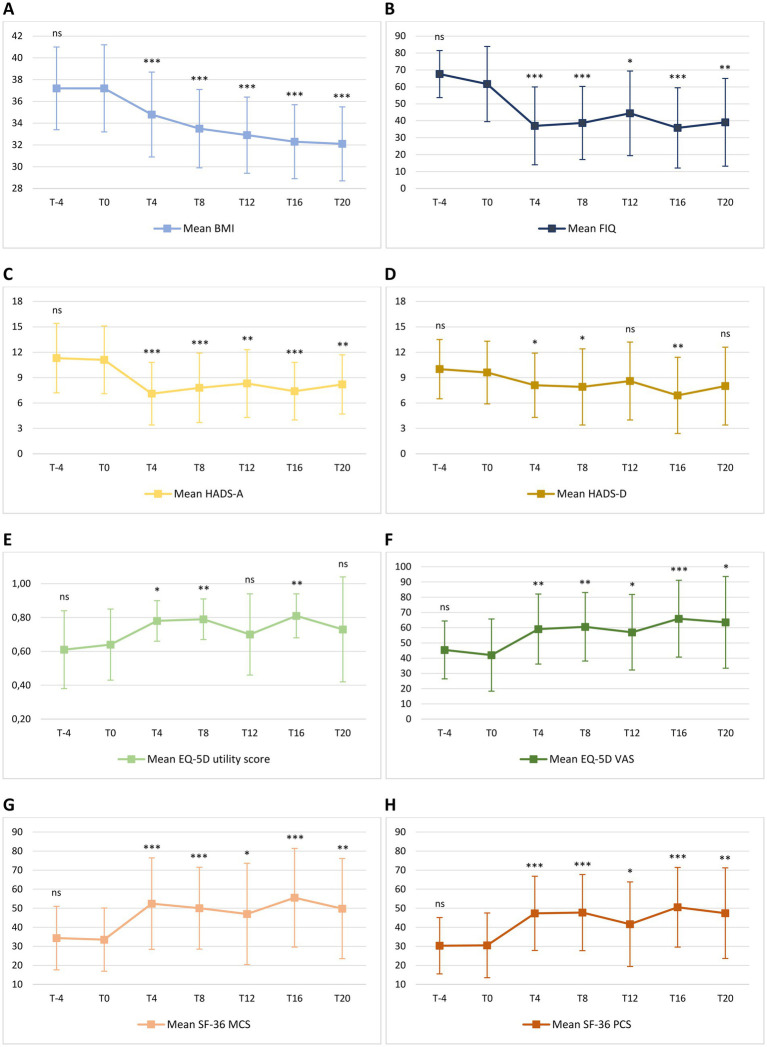
Mean change in body mass index [BMI – **(A)**], Fibromyalgia Impact Questionnaire [FIQ – **(B)**], Hospital Anxiety and Depression Scale [HADS – **(C,D)**], EuroQol 5D [EQ-5D – **(E,F)**] and 36-item Short Form Health Survey [SF-36 – **(G,H)**] from T-4 to T20. Level of significance of each measurement compared with T0 is indicated on the plots: ns = non-significant; * = <0.05; ** = <0.01; *** <0.001. **(A)** Mean BMI was 37.2 ± 3.8 at T-4 and 37.2 ± 4.0 at T0 (*p* = 0.995). During the ketogenic phase, mean BMI significantly decreased to 34.8 ± 3.9 at T4 (*p* < 0.001) and to 33.5 ± 3.6 at T8 (*p* < 0.001). Compared with FIGURE 4 (Continued)baseline values, the decrease in mean BMI was also significant during the carbohydrate reintroduction period, with 32.9 ± 3.5 at T12 (*p* < 0.001), 32.3 ± 3.4 at T16 (*p* < 0.001) and 32.1 ± 3.4 at T20 (*p* < 0.001). **(B)** Mean FIQ was 67.6 ± 13.9 at T-4 and 61.7 ± 22.2 at T0 (*p* = 0.124). Compared to T0, mean FIQ improved during the ketogenic period to 37.0 ± 23.0 at T4 (*p* < 0.001) and to 38.7 ± 21.6 at T8 (*p* < 0.001), with significant changes to 44.4 ± 25.0 observed also at T12 (*p* = 0.012), to 35.8 ± 23.7 at T16 (*p* < 0.001) and to 39.1 ± 25.9 at T20 (*p* = 0.002). **(C)** Mean HADS-A was 11.3 ± 4.1 at T-4 and 11.1 ± 4.0 at T0 (*p* = 0.633). Compared to T0, HADS-A significantly improved to 7.1 ± 3.7 at T4 (*p* < 0.001) and to 7.8 ± 4.1 at T8 (*p* < 0.001). Furthermore, HADS-A decreased to 8.3 ± 4.0 at T12 (*p* = 0.001), to 7.4 ± 3.4 at T16 (*p* < 0.001) and to 8.2 ± 3.5 at T20 (*p* = 0.004). **(D)** Mean HADS-D was 10.0 ± 3.5 at T-4 and 9.6 ± 3.7 at T0 (*p* = 0.571). Compared to T0, mean HADS-D significantly improved to 8.1 ± 3.8 at T4 (*p* = 0.021), to 7.9 ± 4.5 at T8 (*p* = 0.030) and to 6.9 ± 4.5 at T16 (*p* = 0.005). At T12 and T20, a non-significant reduction, respectively, to 8.6 ± 4.6 (*p* = 0.219) and to 8.0 ± 4.6 (*p* = 0.116) was observed. **(E)** Mean EQ-5D utility score was 0.61 ± 0.23 at T-4 and 0.64 ± 0.21 at T0 (*p* = 0.469). Compared to T0, mean EQ-5D utility score significantly increased to 0.78 ± 0.12 at T4 (*p* = 0.010), to 0.79 ± 0.12 at T8 (*p* = 0.003) and to 0.81 ± 0.13 at T16 (*p* = 0.002), while there was no significant difference between the baseline score and the values of 0.70 ± 0.24 (*p* = 0.333) and 0.73 ± 0.31 (*p* = 0.295) observed, respectively, at T12 and at T20. **(F)** Mean EQ-5D VAS score was 45.4 ± 19.0 at T-4 and 42.0 ± 23.7 at T0 (*p* = 0.297). Compared to T0, mean EQ-5D VAS score significantly improved to 59.1 ± 23.0 at T4 (*p* = 0.002), to 60.6 ± 22.5 at T8 (*p* = 0.009), to 57.0 ± 24.8 at T12 (*p* = 0.038), to 65.9 ± 25.2 at T16 (*p* < 0.001) and to 63.5 ± 30.1 at T20 (*p* = 0.022). **(G)** Mean SF-36 MCS was 34.3 ± 16.7 at T-4 and 33.5 ± 16.6 at T0 (*p* = 0.849). Compared to T0, mean SF-36 MCS significantly increased to 52.4 ± 24.0 at T4 (*p* < 0.001), to 50.1 ± 21.5 at T8 (*p* < 0.001), to 47.0 ± 26.6 at T12 (*p* = 0.010), to 55.5 ± 25.9 at T16 (*p* < 0.001) and to 49.8 ± 26.3 at T20 (*p* = 0.002). **(H)** Mean SF-36 PCS was 30.3 ± 14.8 at T-4 and 30.5 ± 17.0 at T0 (*p* = 0.942). Compared to T0, mean SF-36 PCS significantly increased to 47.3 ± 19.5 at T4 (*p* < 0.001), to 47.7 ± 20.0 at T8 (*p* < 0.001), to 41.6 ± 22.2 at T12 (*p* = 0.015), to 50.5 ± 20.9 at T16 (*p* < 0.001) and to 47.4 ± 23.8 at T20 (*p* = 0.009).

Change in mean BMI from T-4 to T20 is shown in Figure 4A. No significant difference in BMI was observed from T-4 to T0 during the free-diet run-in period. Mean BMI was 37.2 ± 3.8 at T-4 and 37.2 ± 4.0 at T0 (*p* = 0.995). During the ketogenic phase, mean BMI significantly decreased to 34.8 ± 3.9 at T4 (*p* < 0.001) and to 33.5 ± 3.6 at T8 (*p* < 0.001). Compared with baseline values, the decrease in mean BMI was also significant during the carbohydrate reintroduction period, with 32.9 ± 3.5 at T12 (*p* < 0.001), 32.3 ± 3.4 at T16 (*p* < 0.001) and 32.1 ± 3.4 at T20 (*p* < 0.001).

#### 3.4.2. Change in FIQ

**Figure 5:**
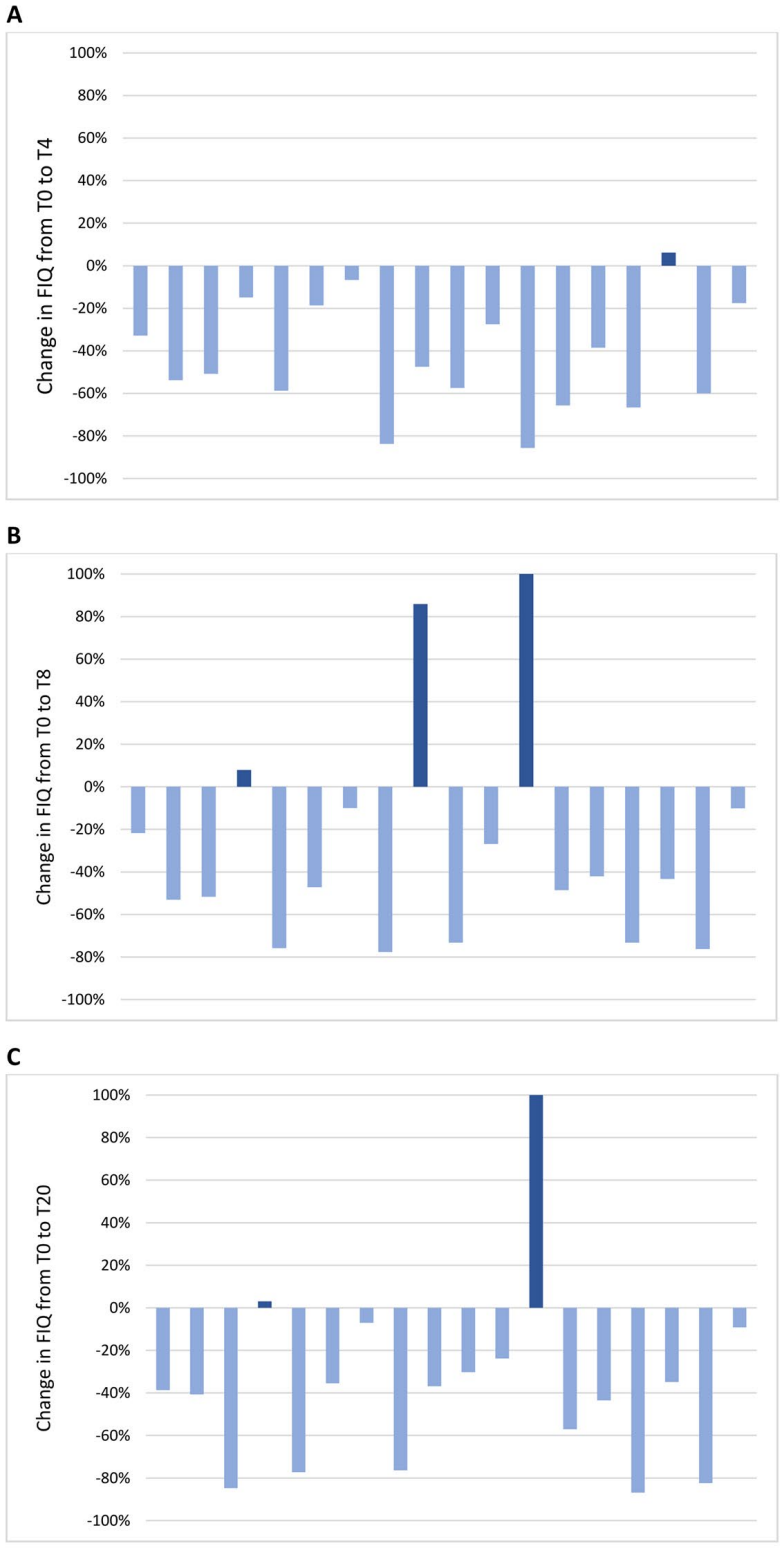
Change in FIQ in individual patients from the baseline visit to T4 **(A)**, T8 **(B)** and T20 **(C)**. **(A)** At T4, FIQ improvement was observed in 17 patients (94%) and it was clinically meaningful in 16 (89%); FIQ worsening was observed in 1 patient (6%) and it was not clinically meaningful. **(B)** At T8, FIQ improvement was observed in 15 patients (83%) and it was clinically meaningful in 13 (72%); FIQ worsening was observed in 3 patients (17%) and it was clinically meaningful in 2 (11%). **(C)** At T20, FIQ improvement was observed in 16 patients (89%) and it was clinically meaningful in 14 (78%); FIQ worsening was observed in 2 patients (11%) and it was clinically meaningful in 1 (6%).

Change in mean FIQ from T-4 to T20 is shown in Figure 4B. No significant difference in mean FIQ was observed from T-4 to T0 during the free-diet run-in period. Mean FIQ was 67.6 ± 13.9 at T-4 and 61.7 ± 22.2 at T0 (*p* = 0.124). Compared to T0, mean FIQ improved during the ketogenic period to 37.0 ± 23.0 at T4 (*p* < 0.001) and to 38.7 ± 21.6 at T8 (*p* < 0.001), with significant changes to 44.4 ± 25.0 observed also at T12 (*p* = 0.012), to 35.8 ± 23.7 at T16 (*p* < 0.001) and to 39.1 ± 25.9 at T20 (*p* = 0.002). Analysing the evolution of FIQ in individual patients during the phase of VLCKD, improvement was observed in 17 patients (94%) at T4 (Figure 5A) and in 15 (83%) at T8 (Figure 5B), and it was clinically meaningful in, respectively, 16 (89%) and 13 cases (72%). Conversely, 2 patients (11%) at T8 experienced a worsening in FIQ exceeding the MCID. FIQ deterioration was driven by an intercurrent SARS-CoV2 infection in one participant and, in the other case, by exacerbation of widespread pain after the transition from the first VLCKD period to the second and third phases when meal preparations were replaced by natural proteins. At the end of the study (Figure 5C), FIQ improvement was still evident in 16 patients (89%) and it was clinically meaningful in 14 (78%).

#### 3.4.3. Change in HADS

Change in mean HADS-A and HADS-D from T-4 to T20 is shown in Figures 4C,D. There was no significant difference in HADS-A and HADS-D from T-4 to T0, during the free-diet run-in period. Mean HADS-A was 11.3 ± 4.1 at T-4 and 11.1 ± 4.0 at T0 (*p* = 0.633). Compared to T0, HADS-A significantly improved to 7.1 ± 3.7 at T4 (*p* < 0.001) and to 7.8 ± 4.1 at T8 (*p* < 0.001). Furthermore, HADS-A decreased to 8.3 ± 4.0 at T12 (*p* = 0.001), to 7.4 ± 3.4 at T16 (*p* < 0.001) and to 8.2 ± 3.5 at T20 (*p* = 0.004).

Mean HADS-D was 10.0 ± 3.5 at T-4 and 9.6 ± 3.7 at T0 (*p* = 0.571). Compared to T0, mean HADS-D significantly improved to 8.1 ± 3.8 at T4 (*p* = 0.021), to 7.9 ± 4.5 at T8 (*p* = 0.030) and to 6.9 ± 4.5 at T16 (*p* = 0.005). At T12 and at T20, a non-significant reduction, respectively, to 8.6 ± 4.6 (*p* = 0.219) and to 8.0 ± 4.6 (*p* = 0.116) was observed.

#### 3.4.4. Change in EQ-5D

Changes in mean EQ-5D utility score and VAS from T-4 to T20 are shown in Figures 4E,F. No significant difference in mean EQ-5D utility score and VAS score was observed from T-4 to T0 during the free-diet run-in period. Mean EQ-5D utility score was 0.61 ± 0.23 at T-4 and 0.64 ± 0.21 at T0 (*p* = 0.469). Compared to T0, mean EQ-5D utility score significantly increased to 0.78 ± 0.12 at T4 (*p* = 0.010), to 0.79 ± 0.12 at T8 (*p* = 0.003) and to 0.81 ± 0.13 at T16 (*p* = 0.002), while there was no significant difference between the baseline score and the values of 0.70 ± 0.24 (*p* = 0.333) and 0.73 ± 0.31 (*p* = 0.295) observed, respectively, at T12 and at T20.

Mean EQ-5D VAS score was 45.4 ± 19.0 at T-4 and 42.0 ± 23.7 at T0 (*p* = 0.297). Compared to T0, mean EQ-5D VAS score significantly improved to 59.1 ± 23.0 at T4 (*p* = 0.002), to 60.6 ± 22.5 at T8 (*p* = 0.009), to 57.0 ± 24.8 at T12 (*p* = 0.038), to 65.9 ± 25.2 at T16 (*p* < 0.001) and to 63.5 ± 30.1 at T20 (*p* = 0.022).

#### 3.4.5. Change in SF-36

Changes in mean SF-36 MCS and PCS from T-4 to T20 are shown in Figures 4G,H. There was no significant difference in mean SF-36 MCS and PCS between T-4 and T0 during the free-diet run-in period. Mean SF-36 MCS was 34.3 ± 16.7 at T-4 and 33.5 ± 16.6 at T0 (*p* = 0.849). Compared to T0, mean SF-36 MCS significantly increased to 52.4 ± 24.0 at T4 (*p* < 0.001), to 50.1 ± 21.5 at T8 (*p* < 0.001), to 47.0 ± 26.6 at T12 (*p* = 0.010), to 55.5 ± 25.9 at T16 (*p* < 0.001) and to 49.8 ± 26.3 at T20 (*p* = 0.002).

Mean SF-36 PCS was 30.3 ± 14.8 at T-4 and 30.5 ± 17.0 at T0 (*p* = 0.942). Compared to T0, mean SF-36 PCS significantly increased to 47.3 ± 19.5 at T4 (*p* < 0.001), to 47.7 ± 20.0 at T8 (*p* < 0.001), to 41.6 ± 22.2 at T12 (*p* = 0.015), to 50.5 ± 20.9 at T16 (*p* < 0.001) and to 47.4 ± 23.8 at T20 (*p* = 0.009).

#### 3.4.6. Association over time between BMI and patient-reported outcomes

All visits between T-4 and T20 of the 18 patients who completed the study period were included in each GEE model, accounting for a total of 126 measurements. The GEE models (Table 3) did not show a significant association between decrease in BMI and change over time of FIQ, HADS-D, EQ-5D utility score or VAS score, SF-36 MCS or PCS. Conversely, the results with HADS-A as dependent variable were significant, suggesting an association between progressive BMI reduction and longitudinal improvement of HADS-A. The β coefficient demonstrated how a patient with a decrease of one point of BMI was expected to have a 0.305 (95% CI 0.039 to 0.570; *p* = 0.024) lower HADS-A.

**Table 3:** Association of BMI and patient-reported outcomes over time.

	β Coefficient (95% CI)	value of *p*
Outcome: FIQ over time		
BMI	0.610 (−0.836 to 2.055)	0.409
Outcome: HADS-A over time		
BMI	0.305 (0.039 to 0.570)	0.024
Outcome: HADS-D over time		
BMI	0.137 (−0.088 to 0.361)	0.233
Outcome: EQ-5D utility score over time		
BMI	−0.003 (−0.018 to 0.120)	0.692
Outcome: EQ-5D VAS score over time		
BMI	−0.636 (−2.754 to 1.481)	0.556
Outcome: SF-36 MCS over time		
BMI	−0.067 (−1.736 to 1.603)	0.938
Outcome: SF-36 PCS score over time		
BMI	−0.241 (−1.568 to 1.087)	0.723

BMI, body mass index; EQ-5D, EuroQoL 5 dimensions; FIQ, fibromyalgia impact questionnaire; HADS-A, hospital anxiety and depression scale-anxiety; HADS-D, hospital anxiety and depression scale-depression; MCS, mental component score; PCS, physical component score; SF-36, 36-item short form health survey; VAS, visual analogue scale.

## 4. Discussion

To our knowledge, this is the first study to investigate the efficacy of VLCKD in FM. The results of our trial show that VLCKD has a rapid and beneficial impact on different functional and psychological domains of the disease. Although a direct comparison is precluded and our study was not designed to address differences between VLCKD and other treatment options, the improvement in FIQ total score resulting from VLCKD was numerically non-inferior to what has been decribed for medications commonly used in FM such as duloxetine, pregabalin or amitriptyline (48–51). Each diet plan was personalized according to the participant’s preferences and the adherence was high, with 90% of patients completing the trial period. Adverse events were mostly mild and transient. Indeed, the restrictive pattern of VLCKD can be challenging for the patients and it is plausible that the adherence was improved by the multidisciplinary medical supervision and the strict counselling provided by experienced rheumatologists, which ensured psychological support to the participants.

Interestingly, all patients lost weight, but we observed a lack of longitudinal association between change in BMI and improvement in PROs over time, with the only exception of HADS-A. This finding suggests that the pleiotropic effects of ketogenic diet on musculoskeletal pain might extend beyond the benefits of weight reduction. Compelling evidence has shown a role of neuroinflammation in the pathogenesis of FM, supported by positron emission tomography studies and by the presence of elevated concentrations of proinflammatory neuropeptides and cytokines in the cerebrospinal fluid of FM patients (52–55). The major ketone bodies beta-hydroxybutyrate and acetoacetate exert their neuroprotective potential mainly through reduction of oxidative stress but also through effects on mitochondria, transcription factors and the composition of gut microbiome (56–58). In the field of neurology, ketogenic diet has an established role in intractable childhood epilepsy and it is increasingly considered as an effective treatment for a wide range of conditions characterized by both refractory seizures and neuroinflammation (59). Therefore, it is conceivable that the positive results observed in our explorative study might be partially explained by the effects of ketone bodies on the modulation of neuroinflammation. Although the consequences of neuroinflammation and the relationship with the key symptoms of FM remain poorly understood, addressing this process may represent a new therapeutic approach in FM patients (60).

Notwithstanding the encouraging findings of our pilot study, some limitations should be recognized. We acknowledge that the sample size was small, only women were included and the duration of the intervention was relatively short, potentially affecting the generalizability of our results to other individuals with FM and especially to male patients. This approach also prevented the possibility of performing post-hoc subgroup analyses on patients with distinct disease characteristics or who achieved more sustained ketosis during the first weeks of the study. The latter analysis, in particular, would have been useful to discriminate the positive effects of weight reduction from the magnitude of improvement contributed by ketosis. The nature of the intervention was unblinded. However, a self-controlled design was implemented to offset the single-arm structure of the study. The run-in phase of free diet preceding the baseline visit provided a 4 weeks reference period in which individuals acted as their own control. No difference in BMI or in any of the assessed outcome measures was observed before the beginning of the VLCKD, suggesting that taking charge of the patient is not sufficient if an effective treatment plan is not initiated. Despite the limitations of this study, several strengths may be mentioned. First, it demonstrates that FM patients can be enrolled in treatment programs that include challenging and highly restrictive nutritional protocols. Second, the diet plan was personalized according to the preferences of each participant, with a variety of meal options and simple recipes, which favoured the high adherence and allowed for the sustained ketosis observed in most cases. Third, we tried to minimize the assessment bias using validated tools which explored different domains of the disease, from pain to daily function, psychological distress and quality of life, which are all factors of particular relevance to FM patients and their families.

In conclusion, in obese women with FM, a 20 weeks program of VLCKD was associated with significant weight reduction and with improvements in all the predefined outcome measures. Given the heterogeneity and complexity of this condition, the positive results and the high retention rate obtained in our study warrant further research. Prospective studies with larger sample and longer follow-up are needed to confirm our observations and to elucidate the potential applications of VLCKD in tailoring the treatment approach to individual FM patients.

## Data availability statement

The raw data supporting the conclusions of this article will be made available by the authors upon reasonable request, without undue reservation.

## Ethics statement

The studies involving human participants were reviewed and approved by Comitato Etico Area Vasta Emilia Centrale. The patients/participants provided their written informed consent to participate in this study.

## Author contributions

JC, LL, AM, SR, GV, and NS contributed to conception and design of the study. LM, VB, and FP organized the database. EA, SiN, and SuN performed the statistical analysis. JC and FP wrote the first draft of the manuscript. AM, LL, LM, VB, CF, and FU wrote sections of the manuscript. All authors contributed to the article and approved the submitted version.

## Funding

Meal replacement preparations were kindly provided by Dieta Medicale (Italy). The funding source had no involvement in the study design, interventions, data collection or interpretation of the results. This research was supported by project 5x1000 year 2020—income 2019 (progetto 5x1000 anno 2020—redditi 2019).

## Conflict of interest

The authors declare that the research was conducted in the absence of any commercial or financial relationships that could be construed as a potential conflict of interest.

## Publisher’s note

All claims expressed in this article are solely those of the authors and do not necessarily represent those of their affiliated organizations, or those of the publisher, the editors and the reviewers. Any product that may be evaluated in this article, or claim that may be made by its manufacturer, is not guaranteed or endorsed by the publisher.
