# Supplementary material for: Efficacy, safety and tolerability of very low-calorie ketogenic diet in obese women with fibromyalgia: a pilot interventional study
Source: Front Nutr. 2023 Jul 12;10:1219321. doi: 10.3389/fnut.2023.1219321 (PMC10369071; doi:10.3389/fnut.2023.1219321)
Supplement: Supplementary file 1 [file Data_Sheet_1.PDF]

FIBROMYALGIA IMPACT QUESTIONNAIRE (FIQ)

Name: ________________________________ Date: / /

Directions: For questions 1 through 11, please circle the number that best describes how you did overall for the *past week*. If you don't normally do something that is asked, cross the question out.

	Always	Most	Occasionally	Never
**Were you able to:**				
Do shopping? ……………………………	*0*	*1*	*2*	*3*
Do laundry with a washer and dryer?	*0*	*1*	*2*	*3*
Prepare meals?	*0*	*1*	*2*	*3*
Wash dishes/cooking utensils by hand?.....	*0*	*1*	*2*	*3*
Vacuum a rug?..	*0*	*1*	*2*	*3*
Make beds?	*0*	*1*	*2*	*3*
Walk several blocks?	*0*	*1*	*2*	*3*
Visit friends or relatives?	*0*	*1*	*2*	*3*
Do yard work?...	*0*	*1*	*2*	*3*
Drive a car?	*0*	*1*	*2*	*3*
Climb stairs?	*0*	*1*	*2*	*3*

12. Of the 7 days in the past week, how many days did you feel good?

0 1 2 3 4 5 6 7

13. How many days last week did you miss work, including housework, because of fibromyalgia?

0 1 2 3 4 5 6 7

(continued)

FIBROMYALGIA IMPACT QUESTIONNAIRE (FIQ) – page 2

Directions: For the remaining items, mark the point on the line that best indicates how you felt overall for the past week.

14. When you worked, how much did pain or other symptoms of your fibromyalgia interfere with your ability to do your work, including housework?

No problem with work ●___I ___I___I ___I___I ___I ___I ___I ___I___● Great difficulty with work

15. How bad has your pain been?

No pain ●___I ___I___I ___I___I ___I ___I ___I ___I___● Very severe pain

16. How tired have you been?

No tiredness ●___I ___I___I ___I___I ___I ___I ___I ___I___● Very tired

17. How have you felt when you get up in the morning?

Awoke well rested ●___I ___I___I ___I___I ___I ___I ___I ___I___● Awoke very tired

18. How bad has your stiffness been?

No stiffness ●___I ___I___I ___I___I ___I ___I ___I ___I___● Very stiff

19. How nervous or anxious have you felt?

Not anxious ●___I ___I___I ___I___I ___I ___I ___I ___I___● Very anxious

20. How depressed or blue have you felt?

Not depressed ●___I ___I___I ___I___I ___I ___I ___I ___I___● Very depressed
