# Supplementary material for: Efficacy, safety and tolerability of very low-calorie ketogenic diet in obese women with fibromyalgia: a pilot interventional study
Source: Front Nutr. 2023 Jul 12;10:1219321. doi: 10.3389/fnut.2023.1219321 (PMC10369071; doi:10.3389/fnut.2023.1219321)
Supplement: Supplementary file 2 [file Data_Sheet_2.PDF]

HOSPITAL ANXIETY & DEPRESSION SCALE (HADS)

Please read each item below and tick the box that comes closest to how you have been feeling this past week. Choose one response from the four given for each interview. Give an immediate response and be dissuaded from thinking too long about the answers.

Name: ____ **Date**: ______________________

DOB: ________________________ **NHS No:** ____________________________________

SCORING

A	**I feel tense or 'wound up**	Most of the time		3
		A lot of the time		2
		Occasionally		1
		Not at all		0

D	**I still enjoy the things I used to enjoy**	Definitely as much		0
		Not quite so much		1
		Only a little		2
		Hardly at all		3

A	**I get a sort of frightened feeling as if something awful is about to happen**	Very definitely & quite badly		3
		Yes, but not too badly		2
		A little, but it doesn't worry me		1
		Not at all		0

D	**I can laugh and see the funny side of things**	As much as always		0
		Not quite so much now		1
		Definitely not so much now		2
		Not at all		3

A	**Worrying thoughts go through my mind**	A great deal of the time		3
		A lot of the time		2
		Not too often		1
		Very little		0

D	**I feel cheerful**	Never		3
		Not often		2
		Sometimes		1
		Most of the time		0

A	**I can sit at ease and feel relaxed**	Definitely		0
		Usually		1
		Not often		2
		Not at all		3

D	**I feel as if I am slowed down**	Nearly all the time		3
		Very often		2
		Sometimes		1
		Not at all		0

HOSPITAL ANXIETY & DEPRESSION SCALE (HADS)

Please read each item below and tick the box that comes closest to how you have been feeling this past week. Choose one response from the four given for each interview. Give an immediate response and be dissuaded from thinking too long about the answers.

Name: ____ **Date**: ______________________

DOB: ________________________ **NHS No**: ____________________________________

A	**I get a sort of frightened feeling like 'butterflies' in the stomach**	Not at all		0
		Occasionally		1
		Quite often		2
		Very often		3

D	**I have lost interest in my appearance**	Definitely		3
		I don't take as much care as I should		2
		I may not take as much care		1
		I take just as much care as ever		0

A	**I feel restless as if I have to be on the move**	Very much indeed		3
		Quite a lot		2
		Not very much		1
		Not at all		0

D	**I look forward to enjoyment to things**	As much as I ever did		0
		Rather less than I used to		1
		Definitely less than I used to		2
		Hardly at all		3

A	**I get sudden feelings of panic**	Very often indeed		3
		Quite often		2
		Not very often		1
		Not at all		0

D	**I can enjoy a good book or radio or TV programme**	Often		0
		Sometimes		1
		Not often		2
		Very seldom		3

**TOTAL SCORES**	**ANXIETY** ___ **DEPRESSION** ___
