# Supplementary material for: Efficacy, safety and tolerability of very low-calorie ketogenic diet in obese women with fibromyalgia: a pilot interventional study
Source: Front Nutr. 2023 Jul 12;10:1219321. doi: 10.3389/fnut.2023.1219321 (PMC10369071; doi:10.3389/fnut.2023.1219321)
Supplement: Supplementary file 3 [file Data_Sheet_3.PDF]

EQ-5D-3L

Health Questionnaire

English version for the UK

(Validated for Ireland)

Sample

© 1990 EuroQol Research Foundation. EQ-5D™ is a trade mark of the EuroQol Research Foundation. UK (English) v2.1

Under each heading, please tick the ONE box that best describes your health TODAY.

MOBILITY

I have no problems in walking about ❑

I have some problems in walking about ❑

I am confined to bed ❑

SELF-CARE

I have no problems with self-care ❑

I have some problems washing or dressing myself ❑

I am unable to wash or dress myself ❑

USUAL ACTIVITIES *(e.g. work, study, housework, family or leisure activities)*

I have no problems with performing my usual activities ❑

I have some problems with performing my usual activities ❑

I am unable to perform my usual activities ❑

PAIN / DISCOMFORT

I have no pain or discomfort ❑

I have moderate pain or discomfort ❑

I have extreme pain or discomfort ❑

ANXIETY / DEPRESSION

I am not anxious or depressed ❑

I am moderately anxious or depressed ❑

I am extremely anxious or depressed ❑

Sample

© 1990 EuroQol Research Foundation. EQ-5D™ is a trade mark of the EuroQol Research Foundation. UK (English) v2.1

- We would like to know how good or bad your health is TODAY.
- This scale is numbered from 0 to 100.
- 100 means the <u>best</u> health you can imagine.
  0 means the <u>worst</u> health you can imagine.
- Please mark an X on the scale to indicate how your health is TODAY.
- Now, write the number you marked on the scale in the box below.

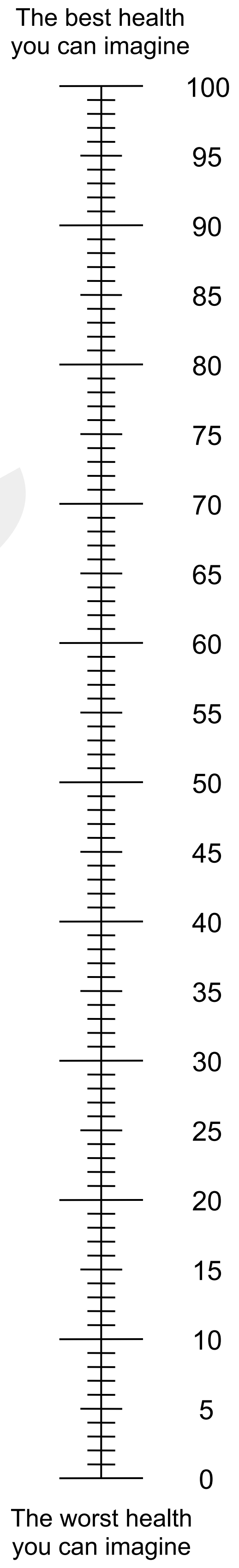

YOUR HEALTH TODAY =

Sample

© 1990 EuroQol Research Foundation. EQ-5D™ is a trade mark of the EuroQol Research Foundation. UK (English) v2.1
